# The Effect of Dual Task on Attentional Performance in Children With ADHD

**DOI:** 10.3389/fnint.2018.00067

**Published:** 2019-01-17

**Authors:** Simona Caldani, Milena Razuk, Mathilde Septier, José Angelo Barela, Richard Delorme, Eric Acquaviva, Maria Pia Bucci

**Affiliations:** ^1^UMR 1141 INSERM, Université Paris Diderot, Hôpital Robert Debré, Paris, France; ^2^EFEE-Centre d’Exploration Fonctionnelle de l’Équilibre chez l’Enfant, Robert Debré Hospital, Paris, France; ^3^Institute of Physical Activity and Sport Sciences, Cruzeiro do Sul University, São Paulo, Brazil; ^4^Child and Adolescent Psychiatry Department, Robert Debré Hospital, Paris, France; ^5^Institute of Biosciences, São Paulo State University, Rio Claro, Brazil; ^6^Paris Diderot University, Paris 7, Paris, France

**Keywords:** eye movements, postural control, dual-task, cerebellum, fixations identification

## Abstract

Attention-deficit/hyperactivity disorder (ADHD) is a common psychiatric disorder without validated objective markers. Oculomotor behavior and executive motor control could potentially be used to investigate attention disorders. The aim of this study was to explore an oculomotor and postural dual task in children with ADHD. Forty-two children were included in the study, gathering children with ADHD (*n* = 21) (mean 8.15 age ± years 0.36) and sex-, age-, and IQ-matched typically developing children (TD). Children performed two distinct fixation tasks in three different postural conditions. Eye movements and postural body sway were recorded simultaneously, using an eye tracker and a force platform. Results showed that children with ADHD had poor fixation capability and poor postural stability when compared to TD children. Both groups showed less postural control on the unstable platform and displayed more saccades during the fixation task. Surprisingly, in the dual unstable platform/fixation with distractor task, the instability of children with ADHD was similar to that observed in TD children. “Top-down” dys-regulation mediated by frontal-striatal dysfunction could be at the origin of both poor inhibitory oculomotor deficits and impaired body stability reported in children with ADHD. Finally, we could assume that the fact both groups of children focused their attention on a secondary task led to poor postural control. In the future it could be interesting to explore further this issue by developing new dual tasks in a more ecological situation in order to gain more insight on attentional processes in children with ADHD.

HIGHLIGHTS

– Children with ADHD showed poor fixation capability when compared to TD children.

– “Top-down” dys-regulation mediated by frontal-striatal dysfunction could be at the origin of both poor inhibitory oculomotor deficits and impaired body stability reported in children with ADHD.

– Both groups of children focused their attention on the visual fixation task leading to poor postural control.

– In the future it could be interesting to develop new dual tasks in an ecological situation in order to gain more insight on attentional processes in children with ADHD.

## Introduction

Attention deficit hyperactivity disorder (ADHD) consists of several abnormal patterns such as inattention, hyperactivity and impulsivity. In Western countries 5% of children are affected by this disorder (American Psychiatric Association, 2013).

In the literature it has been shown that children with ADHD present several deficits in executive motor control and inhibitory abilities (Willcutt et al., 2010). Motor control deficit could be related to a deficit in the integration and processing of different sensory inputs and of visual and vestibular inputs (Wang et al., 2003). A smaller cerebellar vermis which is implicated in the processing of sensory information, as well as in postural control, has been associated to ADHD disorder (Castellanos et al., 1996).

Several studies showed also alterations in the thalamus (Ivanov et al., 2010) and in the cerebellum (Valera et al., 2007) of ADHD patients. Moreover Durston (2003) showed in an fMRI study that children with ADHD presented abnormalities in morphology and functioning of frontostriatal brain circuits. More recently, Hong et al. (2017), using resting state fMRI, studied age-related brain network differences between ADHD patients and typically developing subjects (TD). These authors showed an atypical development in ADHD patients’ brain regions, particularly of left middle temporal gyrus, left inferior frontal gyrus and left insular gyrus. Recall that all these cortical and subcortical networks are involved in performing eye movements and (Leigh and Zee, 2015); indeed poor eye movements performance and inhibitory capabilities had been observed in children with ADHD (see review from Rommelse et al., 2008).

In the domain of eye movements, a fixation task, with or without distractors, involves a strong inhibitory control mechanism. It consists in the ability to maintain an image on the fovea in order to perceive it. It had been also demonstrated that active visual fixation involves a distributed circuitry including frontal eye fields (Goldberg et al., 1986), posterior parietal cortex (Mountcastle et al., 1981; Shibutani et al., 1984), and brain stem structures (Munoz and Wurtz, 1992). Moreover when a distractor is introduced, the activity of the visual fixation system is reduced and the difficulties to maintain engagement to the previous location of the fixation increase (Reilly et al., 2008). Based on these findings, in the present study we wonder to explore further eye movements control, particularly the inhibition capabilities of children with ADHD by testing two different types of fixations calling for different attention load: a simple fixation and a more complex fixation with distractors. Recall that the response inhibition task is known to be a specific task able to discriminate between subjects with ADHD and TD subjects (Tamm et al., 2004), consequently we could expect to find a different oculomotor behavior between ADHD and TD children.

Our aim was to explore postural stability during a dual task (fixation and postural capability) in a group of children with ADHD compared to control children. Several studies from ours and other research groups reported that postural stability is not under an automatic control but it can be influenced by attentional resources, particularly when the secondary task is a complex task (Blanchard et al., 2005; Palluel et al., 2010; Legrand et al., 2013). More recently, our group reported poor postural control during fixation as well as poor quality of fixation itself in children with ADHD (Bucci et al., 2014). However the quality of fixation during simple sitting condition as well as the quality of fixations during a complex fixation with distractor task has not been studied.

It is well known that since attention is involved in the execution of eye movements (Rizzolatti et al., 1987; Deubel and Schneider, 1996) as well as in postural stability (Woollacott and Shumway-Cook, 2002), oculomotor and postural systems are mutually influenced; however, in children with ADHD such relationship could be different given to their poor inhibition/attentional capabilities (Bush, 2010). Indeed, in line with the U-shaped non-linear interaction model of Huxhold et al. (2006) the secondary task executed during a postural task could affect in a different way postural stability that is increasing or decreasing postural sway depending on the complexity of the secondary task. Based on this model, we expect to find an effect of the secondary oculomotor task (fixation task) on postural parameters. Such effect could be different in children with ADHD with respect to TD children according to the amount of attention focused on the secondary oculomotor task. In other way, when the secondary oculomotor task demands less attentional resources (i.e., simple fixation task) we could observe in both children groups tested similar postural as well as oculomotor performance; in contrast, when the secondary oculomotor task is more difficult (i.e., fixation with distractor task) it demands an high attentional load that could decrease postural performance more in children with ADHD than in TD children.

## Materials and Methods

### Participants

#### Clinical Characteristics

Twenty-one children with ADHD (mean 8.15 age ± years 0.36) and twenty-one IQ- and age-matched typically developing children (TD) children (mean age 8.68 ± years 0.34) were recruited at the Child and Adolescent Psychiatry Department, Robert Debré Hospital (Paris, France).

All subjects were evaluated by trained child psychiatrists. The diagnosis of ADHD according to DSM-5 criteria (American Psychiatric Association, 2013) was carried out using the Kiddie-SADS-EP (Goldman et al., 1998). During the general interview related to diagnosis, the presence of psychiatric comorbidities was systematically screened for. The severity of the ADHD symptoms was estimated by the ADHD Rating Scale-parental report (ADHD-RS). All children with ADHD were also assessed using the Wechsler scale (Wechsler Intelligence Scale for Children, fourth edition), the Beery-Buktenica Developmental Test of Visual-Motor Integration (VMI, Beery, 1997) and the Motor Assessment Battery for Children (MABC) (Henderson and Sugden, 1992).

To confirm the absence of ADHD, controls were systematically interviewed. In order to be included in our study, controls needed to have a total score ≤10 on the ADHD-RS (Dickson et al., 2011) and a neurological examination in the normal range.

Moreover IQ was evaluated in controls using two subtests, one related to verbal ability (the similarities test) and the other related non-verbal abilities (matrix reasoning test).

The score on these two tests was not significantly different between the two groups of children. The clinical characteristics of children with ADHD and controls are reported in Table 1.

**Table 1:** Clinical characteristics of ADHD children and typically developing children (TD) tested.

	TD	ADHD
	*N* = 21	*N* = 21
**Clinical data**		
Age (years)	8.68 ± 0.34	8.15 ± 0.36
**ADHD-RS**		
ADHD-RS total score	4 ± 0.8	37 ± 2
ADHD-RS inattention subscore	-	17.9 ± 1.4
ADHD-RS hyperactivity/impulsivity subscore	-	18.3 ± 1.7
**Wechsler scale (WISC-IV) scores**		
Verbal Comprehension subscale	-	99.1 ± 3.3
Perceptual Reasoning subscale	-	93.6 ± 3.5
Working Memory subscale	-	86.6 ± 3.0
Processing Speed subscale	-	90.9 ± 2.7
Similarity test	10.06 ± 0.4	9.9 ± 0.6
Matrix reasoning test	10.14 ± 0.5	9.7 ± 0.5

The investigation adhered to the principles of the Declaration of Helsinki and was approved by our Institutional Human Experimentation Committee (Comité de Protection des Personnes CPP, Ile de France V, Hôpital Saint-Antoine).

After the nature of the procedure had been explained, a written informed consent was obtained from the participants and their parents.

#### Visual Conditions

**FIGURE 1:**
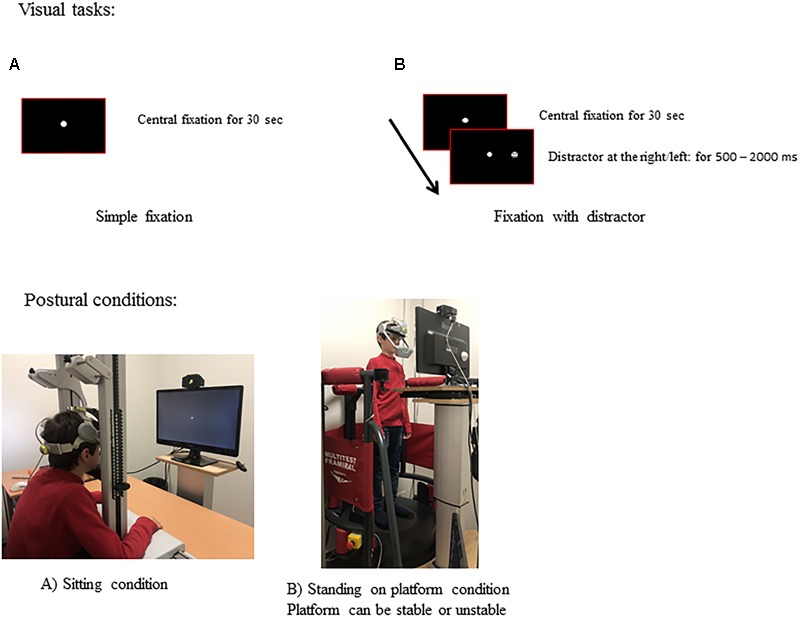
Experimental set-up. Visual tasks and postural conditions used. Temporal arrangement of *simple fixation* **(A)** and of *fixation with distractor* **(B)** task. Example of a child in sitting condition **(A)** and in standing on platform condition **(B)**. Note that in standing on platform condition, than platform was stable and unstable (it was moved by oscillations allowing forward and backward translations).

All children performed two visual tasks paradigms. In the first one, named *simple fixation* (see Figure 1A), the child was invited to fixate on the target appearing in the center (filled white circle subtending a visual angle of 0.5°) of the black screen during 30 s. In the second one, named *fixation with distractors* (see Figure 1B), the child had to maintain fixation on the central target and to inhibit saccades toward the distractors. The distractor was a white smile target (of 0.5°) appearing for a random duration from 500 to 2000 ms and calling for horizontal saccade amplitudes from 5° to 20°. The distractor was presented during the *fixation with distractor* trail (30 s), in total 8 distractors were presented during each *fixation with distractor* trial. Instructions were given to the child to try to fixate the central target as better as possible (in the *simple fixation* task) and to try to fixate the central target as better as possible without looking the distractors (in *the fixation with distractor* task).

#### Postural Conditions

The experimental sessions took place in a dark room. Three postural conditions were performed (see Figure 1).

*Simple sitting condition:* Children were asked to sit in a comfortable chair. Their head was stabilized by a forehead and chin support while their eye movements were recorded.

*Complex standing on stable platform condition:* Children were asked to stand upright on the Framiral^®^stable platform with their arms along their body and their feet on the footprints.

*Complex standing on unstable platform condition:* Children were asked to stand upright on the Framiral^®^unstable platform with their arms along their body and their feet on the footprints.

Eye movements and body sway were recorded simultaneously.

For these three postural conditions, the PC monitor, in which the two visual tasks were presented, was placed 60 cm away and adjusted at each child’s eye level (see Figure 1). Each visual task (*simple fixation* and *fixation with distractor*), respectively for each of the three postural conditions tested was performed twice, with the order of each trial defined randomly. The number of saccades made during fixation task and the postural measures from the same visual and postural conditions tested for each child were averaged.

#### Eye Movement Recording

Fixation performance was recorded by the Mobile EBT Tracker (SuriCog), a CE-marked medical eye-tracking device. The Mobile EBT is equipped with cameras that capture the movements of each eye independently. The frequency of recording was set up to 300 Hz and system precision was 0.25°. There was no obstruction of the visual field with this recording system.

#### Postural Recording

The excursions of the center of pressure (CoP) were recorded with Multitest Equilibre (Framiral^®^, Grasse, France), also called Balance Quest. This device consists in a platform which can be stable or unstable. In case of unstable condition the platform can be moved by oscillations allowing forward and backward translations, with a constant linear velocity which may vary from 0.03 to 0.07 m/s with a frequency of 0.25 Hz.

The displacement of the CoP was sampled at 40 Hz and digitized with 16-bit precision. Postural recording was performed in stable and unstable platform conditions. The duration of each postural condition was of 30 s. Visual task started at the same time of postural recording lasting 30 s.

#### Data Analysis

Calibration factors for each eye were determined from the eye positions during the calibration procedure (see Bucci and Seassau, 2012) done before the visual task. Eye movement analyses were performed using the MeyeAnalysis software, which automatically determined the onset and the end of each saccade by using a built-in saccade detection algorithm, and visually inspected and verified by the investigator. For both visual conditions (simple fixation and fixation with distractor) we counted the number of saccades done during the paradigm. All saccades ≥2° were counted given that it is well known that micro-saccades are normally of smaller amplitude (for more details, see Tiadi et al., 2016). The time looking outside the fixation target in teh two visual tasks was also evaluated.

Postural control performance was evaluated using the surface area of the CoP (in cm^2^) and the mean velocity (mm/s). The surface of the CoP was calculated corresponding to the area of an ellipse encompassing 90% of all CoP data point excursions.

#### Statistical Analysis

After testing the normality and homogeneity of variance assumptions on eye movements results a three-way ANOVA with children (ADHD and TD), visual task (fixation and fixation with distractor), and postural condition (sitting, standing stable, and standing unstable) as factors was run. In contrast, on postural measure two-way ANOVA was run with children (ADHD and TD), visual task (*simple fixation* and *fixation with distractor*) and postural condition (standing stable and standing unstable) as factors. Note that postural stability was not measured during the sitting condition. When necessary, Bonferroni *post-hoc* comparisons were employed. Finally, the Student paired *t*-test was also done comparing the surface area and in the mean velocity of the CoP of the two postural conditions (stable and unstable platform) for the two groups of children. Analyses were performed using the Statistica software the GLM (Advanced Linear Models) software and the level of significance was kept at 0.05.

## Results

### Eye Movements

**FIGURE 2:**
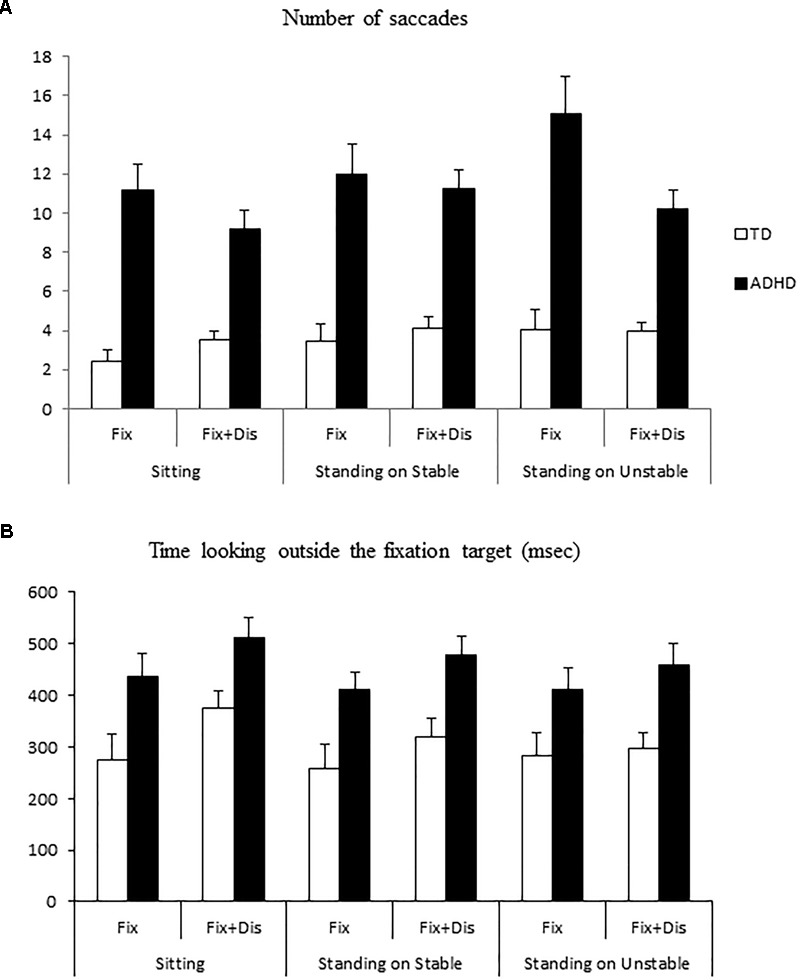
**(A)** Means and standard deviations of number of saccades in three different postural conditions (sitting, standing on stable, and unstable platform) in typically developing and attention-deficit/hyperactivity disorder (ADHD) children. **(B)** Means and standard deviations of the time looking outside the fixation target or at the distractor in typically developing and ADHD children.

Figure 2A shows the number of saccades made by the two groups of children in the two different visual tasks during the three postural conditions. The ANOVA showed that during fixation tasks children with ADHD made significantly more saccades compared to TD children [*F*_(1,41)_ = 94, *p* < 0.0001].

ANOVA failed to show a significant effect of visual task and of postural condition [*F*_(1,41)_ = 2.44, *p* = 0.1 and *F*_(1,41)_ = 2.36, *p* = 0.1, respectively], but it reported a significant postural and visual task interaction [*F*_(2,82)_ = 4, *p* < 0.05]. *Post hoc* tests showed that both groups of children made a larger number of saccades in the simple fixation task during standing condition on the unstable platform with respect to both visual tasks in simple sitting condition (*p* < 0.02 and *p* < 0.003, respectively). Moreover, during the standing condition on the unstable platform, both groups made more saccades than in the standing condition on the stable platform (*p* < 0.004).

ANOVA also showed significant group and visual task interaction [*F*_(1,41)_ = 15.070, *p* < 0.0001]. *Post hoc* tests showed that the number of saccades in both visual tasks was higher for children with ADHD when compared to TD children (both *p* < 0.0001). Moreover, *post hoc* tests showed that children with ADHD made more saccades during the simple fixation task than during the fixation with distractors task (*p* < 0.002).

Figure 2B shows the time looking outside the fixation target or at the distractor for the two groups of children tested. ANOVA showed a significant effect of group [*F*_(1,41)_ = 18.24, *p* < 0.0004]: children with ADHD looked more time outside the fixation target. ANOVA failed to show any significant effect of visual task and of postural condition.

### Postural Control

**FIGURE 3:**
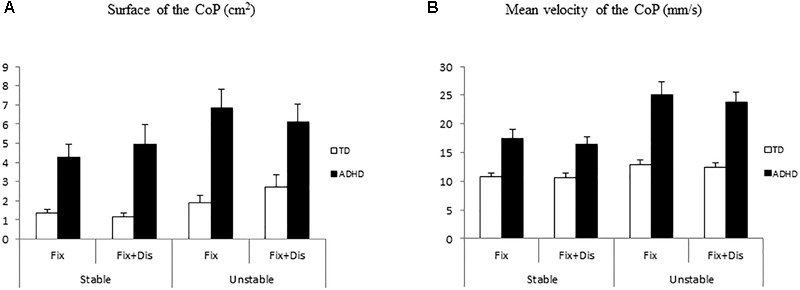
Means and standard deviations of surface **(A)** and mean veloticy **(B)** of the CoP in two different postural conditions (standing on stable and unstable platform) in typically developing and ADHD children.

Concerning postural conditions, ANOVA showed a significant effect of group on the surface of the CoP [*F*_(1,41)_ = 20.86, *p* < 0.0001]. The surface of the CoP was significantly larger in children with ADHD than in TD children (see Figure 3A). Moreover, ANOVA showed significant effect of postural condition [*F*_(1,41)_ = 8.18, *p* < 0.006], with the surface of the CoP significantly larger when standing on the unstable platform than on the stable platform; in contrast ANOVA failed to show any significant effect of visual task [*F*_(1,41)_ = 0.31, *p* = 0.5].

Finally, ANOVA showed significant group, postural and visual task interaction [*F*_(1,41)_ = 5.6, *p* < 0.002]. *Post hoc* tests showed that ADHD children in simple fixation task while standing on the unstable platform had larger surface of CoP with respect to all the other conditions reported in children with ADHD as well as in TD children (all *p* < 0.0001). Moreover, *post hoc* test showed that the surface of the CoP for children with ADHD in the fixation with distractor task in the unstable postural condition was significantly larger with respect to TD children (all *p* < 0.04) in all but one condition (fixation with distractor on the unstable platform); surface of the CoP of children with ADHD was also significantly larger than those reported in this group of children in simple fixation task on the stable platform (*p* < 0.0002).

Concerning mean velocity of the CoP (see Figure 3B), ANOVA showed a significant effect of group: ADHD children had larger mean velocity in contrast to TD children [*F*_(1,41)_ = 32.04, *p* < 0.0001]. Moreover, ANOVA showed significant effect of postural condition: standing on the unstable platform the mean velocity was larger than standing on the stable platform [*F*_(1,41)_ = 41.76, *p* < 0.0001], while it failed to show any significant effect of visual task [*F*_(1,41)_ = 3.22, *p* = 0.08].

Finally, ANOVA showed group and postural condition interaction [*F*_(1,41)_ = 16.19, *p* < 0.0001]. *Post hoc* tests showed that children with ADHD standing on the stable platform had larger mean velocity than TD children in the same postural condition (*p* < 0.008). The mean velocity in children with ADHD standing on the unstable platform was also significantly larger with respect to TD children in both postural conditions and to children with ADHD standing on the stable platform (all *p* < 0.0001).

Finally, we wonder to explore further whether the increase of the surface area and of the mean velocity of the CoP was more important in ADHD group compared to TD group. For TD children both the surface area and the mean velocity of the CoP were significantly larger in unstable condition (*t* = –2.56, *p* < 0.01, and *t* = -2.78, *p* < 0.01, respectively), while for children with ADHD the mean velocity of the CoP only was significantly larger in unstable condition (*t* = -5.6, *p* < 0.0001) but the surface area of the CoP was similar in both stable and unstable condition (*t* = -1.9, *p* = 0.6).

## Discussion

The aim of this study was to explore oculomotor and postural dual tasks in children with ADHD.

The present study reports that: (i) children with ADHD have poor fixation capabilities when compared to TD children; (ii) children with ADHD show more difficulties during simple fixation visual task than in fixation with distractors task; (iii) children with ADHD show poor postural stability compared to TD children; (iv) all children had more difficulty standing on the unstable platform (the surface area of the CoP was significantly larger); and (v) both groups of children made more saccades during simple fixation on the unstable platform than in the sitting condition; interestingly, on the unstable platform during the simple fixation task, children with ADHD were significantly more unstable than TD children, while in fixation with distractor task, the surface of the CoP was similar for the two groups of children. Each finding will be discussed below.

### Poor Fixation Capabilities in Children With ADHD

Our results show that children with ADHD made more saccades than TD children, particularly during the simple fixation task. These findings are in line with the literature and suggest that children with ADHD have a difficulty of inhibitory control mechanism (Gould et al., 2001; Feifel et al., 2004; Bucci et al., 2017). A high number of saccades during fixation could suggest that these children have difficulty to maintain fixation, which is consistent with a failure of “top-down” regulation mediated by frontal-striatal dysfunction (Gould et al., 2001) given that these regions are in fact implicated in visual fixation activity (Wurtz and Goldberg, 1989).

Indeed, neurophysiological studies on saccades have shown that Frontal Eye Fields has two principal connections with the Superior Colliculus, namely a direct pathway (fronto-tectal) and an indirect one via basal ganglia (Hikosaka et al., 2000). Based on these findings, we suggest that children with ADHD could have abnormalities in this cortical/central network. Indeed, in a meta-analysis report based on fifty-five fMRI studies (Cortese et al., 2012), hypoactivation has been observed in frontoparietal and ventral attention networks in children with ADHD when compared to controls.

Furthermore, in this study we observed that children with ADHD made more saccades during the simple fixation task compared to the fixation with distractors task; one could make the hypothesis that this could be due the fact that children with ADHD were looking more at the distractor leading to make less saccades in this specific visual condition, but this was not the case given that the time spended by looking outside the fixation target was similar in both visual tasks (simple fixation as well as fixation with distractors). This finding is quite surprising and it is in contrast to our pervious hypothesis, according to which children with ADHD having poor inhibitory capabilities (Feifel et al., 2004; Bucci et al., 2017) could make more saccades in the fixation with distractor task with respect to the simple fixation task.

We could explain this finding by the fact that children with ADHD are able to orient their attention to the more difficult visual task (as the fixation with distractor). Indeed, according to the multiple-resources model of attention (Wickens, 1991) child would use in a different way attentional resources depending on the type of the task he/she has to perform. In more details, if the two tasks are of similar type (i.e., visual information), as in fixation with distractor task in which the child has to fixate a target and at the same time to avoid to fixate the distractors, he/she has difficulties to focus attention on one of the two visual tasks because they interfere each other. In contrast, children with ADHD who have attentional and inhibitory deficiencies could focus better their attention in such conditions where the interference level and attentional load is higher leading to a better performance of this visual task (fixation with distractor task). In other words, if attentional demanding is high, children with ADHD could increase their attentional performance; this finding need to be tested further by using dual task of similar type with higher level of interference (i.e., attention).

### Poor Postural Performance in Children With ADHD

In this study, we have found that children with ADHD have a poorer postural control than that of TD children. This is also showed by the fact that the surface area only and not the mean velocity of the CoP was similar in children with ADHD independently to the postural condition (stable/unstable). Such finding could suggest that children with ADHD make more muscular effort (shown by high velocity of the CoP) to try to reduce their body sway.

Our finding is in line with previous studies. Recall that motor deficiencies occur in 30–50% of children with ADHD (Goulardins et al., 2017); ADHD is frequently associated with poor gross and fine motor control capabilities (Piek et al., 1999; Wang et al., 2011; Papadopoulos et al., 2014) and our group (Bucci et al., 2014, 2016) as well as other groups (Zang et al., 2002; Wang et al., 2003; Buderath et al., 2009) showed postural instability in ADHD children compared to control children. In line with these studies, we can suggest a deficit in sensory processing for body stability in central structures. More recently, Kim et al. (2017), evaluating postural and gait balance as well as functional connectivity of brain regions controlling balance, reported that children with ADHD showed disturbances of balance and posture that could be associated to decreased brain connectivity in the premotor cortex. Particularly, they showed an alteration of connectivity from the cerebellum to the middle frontal gyrus and medial frontal gyrus in children with ADHD with respect to control children.

### Dual Task Effect

Firstly, our study reported that both groups of children showed a better quality of fixation when they were sitting than when they were on the unstable platform; this could be due to the fact that when postural load is high, children shift their attention on postural control, leading to poor fixation performance. However, we also found that when both tasks (oculomotor and posture) became more difficult (fixation with distractor on unstable platform) the instability in terms of the surface of the CoP of children with ADHD was similar to that of TD children. This finding is in line with the model of U-shaped non-linear interaction of Huxhold et al. (2006), according to which secondary task can influence postural stability differently. However, this could also be due to the “ceiling effect”: the dual task requires a high level of attention resources that cannot be allocated to posture by both groups of children. This finding is in line with the study of Shorer et al. (2012), showing that the effect of dual task on postural performance was similar in children with ADHD and TD children. Moreover, Manicolo et al. (2017) explored the dual task effect of walking and they did not observe any difference in gait performance during a dual cognitive task. Based on all these studies, we could assume that both groups of children (ADHD and TD) prioritized the fixation task over the postural task that is more difficult in unstable condition, in agreement with the U-shaped non-linear interaction model of Huxhold et al. (2006).

Note, however, that small head movements could occur during stable/unstable postural conditions and that unfortunately, we do not have already a system able to measure at the same time both head and eye movements. In the future it could be interesting to explore further this issue by developing a new dual task in a more ecological situation by recording eye/head/body movements in order to gain more insight on how children with ADHD can focus their attention.

Finally, the evaluation of oculomotor and postural dual task could be considerated as a potential biomarker allowing a better discrimination between subjects with ADHD with respect to the subjects with neurodevelopmental disorders (for example with autism spectrum disorder, ASD). In fact subjects with ASD showed more executive dysfunctions such as planification and flexibility (Hill, 2004), consequently we could expect that children with ASD would perform this type of dual task worse than children with ADHD. However further studies need to be done in order to better define the salient characteristics of each of these two diseases.

## Conclusion

Our data suggest that children with ADHD have difficulty of inhibitory control mechanism and poor postural stability, probably due to a failure of “top-down” regulation mediated by frontal-striatal dysfunction.

Finally, we could assume that both groups of children focused their attention on an oculomotor task, leading to poor postural control.

In the future it could be interesting to test whether postural training could improve attentional performance in children with ADHD.

## Author Contributions

MB acquired funding and conceptualized, supervised, wrote, and reviewed and edited the manuscript. SC and MR curated the data, analyzed formal, and reviewed and edited the manuscript. MS and EA curated the data and reviewed and edited the manuscript. JB and RD reviewed and edited the manuscript.

## Conflict of Interest Statement

The authors declare that the research was conducted in the absence of any commercial or financial relationships that could be construed as a potential conflict of interest.
